# Classification of *CHD7* Rare Variants in Chinese Congenital Hypogonadotropic Hypogonadism Patients and Analysis of Their Clinical Characteristics

**DOI:** 10.3389/fgene.2021.770680

**Published:** 2022-01-03

**Authors:** Bang Sun, Xi Wang, Jiangfeng Mao, Zhiyuan Zhao, Wei Zhang, Min Nie, Xueyan Wu

**Affiliations:** NHC Key Laboratory of Endocrinology (Peking Union Medical College Hospital), Department of Endocrinology, Peking Union Medical College Hospital, Peking Union Medical College, Chinese Academy of Medical Sciences, Beijing, China

**Keywords:** congenital hypogonadotropic hypogonadism, CHARGE syndrome, *CHD7* variants, variant spectrum, phenotype spectrum

## Abstract

**Purpose:** *CHD7* rare variants can cause congenital hypogonadotropic hypogonadism (CHH) and CHARGE syndrome. We aimed to summarize the genotype and phenotype characteristics of CHH patients with *CHD7* rare variants.

**Methods:** Rare sequencing variants (RSVs) were detected by Sanger sequencing in a series of 327 CHH patients and were interpreted and grouped according to the American College of Medical Genetics and Genomics (ACMG) guideline. Detailed phenotyping and genotype-phenotype correlation were analyzed.

**Results:** The RSV detection rate was 11.01% (36/327) in the CHH patients. We identified 30 RSVs and 19 of them were novel. Following ACMG criteria, three variants were pathogenic (P), 4 were likely pathogenic (LP), 3 were of uncertain significance with paradoxical evidence (US1), and 20 were of uncertain significance without enough evidence (US2). All patients (4/4, 100%) with P or LP variants manifested extragonadal symptoms.

**Conclusion:** Addition of 19 novel *CHD7* variants expanded the spectrum of variants, and pathogenic or likely pathogenic RSVs were more likely to cause syndromic CHH. For CHH patients carrying *CHD7* RSVs, detailed genotyping and phenotyping can facilitate clinical diagnosis and therapy.

## Introduction

The hypothalamic-pituitary-gonadal axis is indispensable to human puberty and fertility (Riecher-Rössler, 2017; Herbison, 2016). A deficiency of gonadotropin-releasing hormone (GnRH) results in congenital hypogonadotropic hypogonadism (CHH, OMIM 146110)—a rare genetic disorder [1 in 30,000 males and 1 in 125,000 females (Laitinen et al., 2011)] featuring incomplete or absent puberty and infertility. If anosmia co-exists, which occurs in about 50% cases (Bhagavath et al., 2006; Seminara et al., 1998; Stamou and Georgopoulos, 2018; Seminara et al., 1998; Bhagavath et al., 2006; Stamou and Georgopoulos, 2018), the condition is described as Kallmann syndrome (KS), otherwise it is considered normosmic congenital hypogonadotropic hypogonadism (nCHH). The clinical and genetic manifestations of CHH are heterogeneous; more than 40 pathogenic genes have been identified, accounting for about 50% of the pathogenesis (Seminara et al., 1998; Bhagavath et al., 2006; Stamou and Georgopoulos, 2018).

The *CHD7* gene, which is located in chromosome 8q12 and spans 188 kb (Vissers et al., 2004), consists of 38 exons and encodes chromodomain helicase DNA binding protein 7 (CHD7), a 2997-amino acid protein including nine functional regions. CHD7 is the first identified chromatin-remodeling protein contributing to human puberty; previous studies have proved its critical role in maintaining GnRH level and olfactory neuron maturation (Kim et al., 2008). Its structure is highly conserved, and it is expressed ubiquitously in the human body. Pathogenic variants of *CHD7* exist in about 10% of CHH patients, and they are also the major pathogenic cause (detection rate is more than 90%) for another autosomal dominant disease, CHARGE syndrome (OMIM, 214800) (Bergman et al., 2011). The acronym CHARGE stands for Coloboma of the eye, Heart defects, Atresia of the choanae, Retardation of growth/development, Genital abnormalities, and Ear abnormalities (Martin, 2010). According to Verloes’ diagnostic criteria (Verloes, 2005), there are eight key clinical items, including three major items (coloboma, choanal atresia, semicircular canal anomalies) and five minor items (rhombencephalic anomalies, hypothalami-hypophyseal dysfunction, external or middle ear malformations, malformation of mediastinal viscera, and intellectual disability). Based on the number of major and minor items, patients were classified into three groups: typical, partial, and atypical CHARGE. Besides CHH and CHARGE syndrome related symptoms, *CHD7* variants can also lead to other CHD7-related manifestations, including abnormalities of the skeleton, muscle, skin, digestive system and urinary system.

Growth retardation and genital abnormalities are potential overlapping symptoms between CHARGE and CHH. Missense *CHD7* variants are more common in CHH patients, whereas null variants (e.g., nonsense, frameshift) are more common in CHARGE syndrome (Balasubramanian et al., 2014). However, the specific correlation between the two diseases has not been clarified owing to the lack of detailed variant annotation and large clinical trials. In this study, we aimed to explore the genotype, phenotype and genotype-phenotype correlation of CHH patients with *CHD7* rare sequencing variants (RSVs). We analyzed *CHD7* RSVs in a series of 327 Chinese CHH patients, classified them according to the American College of Medical Genetics and Genomics (ACMG) guideline, evaluated their clinical characteristics (focused on CHARGE-related symptoms and other CHD7-related ones), and performed genotype-phenotype correlation analysis.

## Methods

### Participants and Inclusion/Exclusion Criteria

Three hundred and fifty-seven probands were admitted to Peking Union Medical College Hospital (Beijing, China) between January 2005 and December 2012 and diagnosed as CHH. This study included 327 unrelated CHH Chinese probands: 148 nCHH (141 males; Seven females) and 179 KS (167 males; 12 females), excluding 30 patients diagnosed as carrying *FGFR1* variants (Nie et al., 2021). The study was approved by the Ethics Committee for Human Research of Peking Union Medical College Hospital; all patients and guardians of children provided written informed consent.

The inclusion and exclusion criteria were laid down based on CHH standard consensus (Boehm et al., 2015). Inclusion criteria: (1) absent or incomplete puberty by the age of 18 years for male and 16 years for female; (Herbison, 2016) cryptorchidism without a definite diagnosis; (2) serum testosterone≤100 ng/dl for males or estradiol≤20 pg/ml for females, with low or normal serum gonadotropin level. The participants were classified as having KS; (3) when the inquiry information showed the presence of anosmia or hyposmia, otherwise they were classified as having nCHH. Exclusion criteria: diagnosis of secondary hypogonadotropic hypogonadism induced by tumors, trauma, drugs, or other systemic diseases.

### Variants Screening

We collected blood samples from all participants. Focusing on the *CHD7* gene, the Sanger sequencing technique was used to detect causative variants. Genomic DNA extraction was performed using the QIAGEN Midi Blood kit (QIAGEN, Germany) from leukocytes in peripheral blood. Polymerase chain reaction (PCR) was used to amplify the specific sequence of *CHD7* gene. All experiments followed the manufacturer’s instructions.

We read the sequencing results by 4 Peaks (Nucleobytes, Netherlands), then blasted each in NCBI (https://blast.ncbi.nlm.nih.gov/Blast.cgi), against the following reference sequences: NG_007009 (g.DNA); NM_017780 (c.DNA); NP_060250 (p.Protein). Considering the digenic or oligogenic pathogenic possibility, which may explain why individuals with the same *CHD7* variant presented with different phenotypes, we screened and annotated the other CHH-related genes by Sanger sequencing, including *ANSO1*, *FGF8*, *PROK2*, *PROKR2*, *GNRH1*, *GNRHR*, *KISS1*, *KISS1R*, *TAC3*, *TACR3*, *LEP, LEPR*, *NELF*, *WDR11*, *HS6ST1*, and *SEMA3A*. All variants were reported according to Human Genome Variation Society (HGVS) nomenclature rules (Dunnen and Antonarakis, 2000).

### Annotation for Identified Variants

**FIGURE 1:**
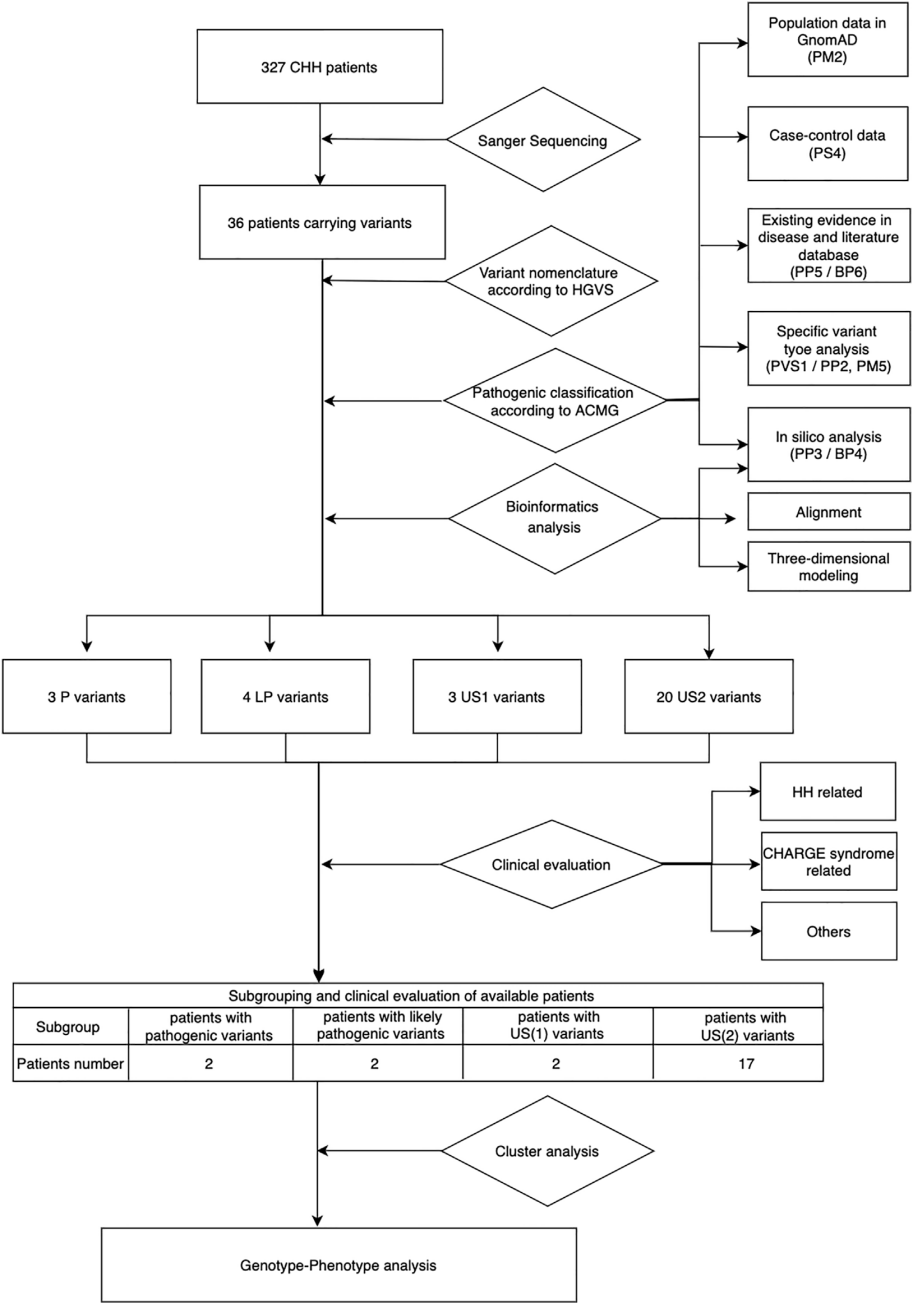
Workflow of genetic and phenotypic analysis. US1, uncertain significance with paradoxical evidence; US2, uncertain significance without enough pathogenic evidence.

Based on the ACMG standards and guidelines^3^, we filtered all detected variants step by step. The core workflow includes general population database search, case-control statistical comparison based on calculation of odds ratio (OR) and the 95% confidence interval (95%CI) (referring to the statistical analysis section for details), disease database search, literature query, variant-type-specific analysis, and computational prediction (Figure 1). We used the allelic frequency from gnomAD database to calculate OR and define a rare sequencing variant (RSV) [a maximum allele frequency (MAF) < 0.0001]. Variants databases from patients included ClinVar and *CHD7* databases. Literature query was performed in PubMed, Google Scholar and China National Knowledge Infrastructure (CNKI). The search key word included “*CHD7* gene,” “hypogonadotropic hypogonadism” and related variants without any language, timeframe or article type restriction.

Computational prediction included three parts: pathogenic prediction by in silico tools, conservation analysis by alignment, and three-dimensional visualization by three-dimensional modeling software. In silico tools included five [SIFT (Sim et al., 2012), Polyphen-2 (Adzhubei et al., 2010), SNP&GO (Schwarz et al., 2010), Mutation Assessor (Reva et al., 2011), and Mutpred (Li et al., 2009)] for missense pathogenicity prediction (when more than three predicted the variants as pathogenic, the evidence pointed to PP3, otherwise it was BP4) and two [Splice Site Score Calculation and SpliceAI (Jaganathan et al., 2019)] for splicing sites (Table 2). We judged the evolutionary conservation of each amino acid site by aligning the human *CHD7* sequence with orthologs of other 25 related species in UniProt database with Clustal W; the more conservative the site is, the more important it may be for protein function, which indirectly reveals its pathogenicity. Amino acid change was visualized by ChimeraX, a three-dimensional modeling software. By comparing the change in the identity of wild and mutant amino acids and their contact with other amino acids in a spatial model, we can predict the potential pathogenic effects the specific variant brings to the protein.

Data for all pathogenic and benign variants considering the above aspects were collected when available, and then based on the weight of the evidence, we classified the variants into six groups: pathogenic (P), likely pathogenic (LP), uncertain significance with paradoxical evidence (US1), uncertain significance without enough pathogenic evidence (US2), likely benign (LB), and benign (B). All specific criteria above were based on the ACMG guideline, except that we further divided the US ones into US1 and US2 based on the type of evidence (paradoxical evidence or insufficient pathogenic evidence).

### Clinical Evaluation

We recorded basic information of all CHH patients during their first visit, including sex, age, height, weight, testicular size, penis length, hormone levels, and olfactory magnetic resonance imaging (MRI) results, medical history and family history. Prader orchidometer was used to measure the testicular size, and the mean volume was calculated for statistical analysis. Hormone tests results included levels of serum follicle-stimulating hormone (FSH), luteinizing hormone (LH), and testosterone (T), which were measured by chemiluminescent immunoassays (Bayer Diagnostics Corporation, United States).

For CHH patients with *CHD7* RSVs, detailed phenotyping was performed when possible. Clinical evaluation items were based on the Human Phenotype Ontology database and were further classified into three types: CHH-related, CHARGE syndrome-related (including the function and appearance of eyes, nose, ears, mouth, and face; growth status, intelligence, and heart condition), and others (including the condition of the skeleton, muscle, palatal arch, skin, digestive system, and urinary system and synkinesia). The CHARGE-related symptoms were based on Verloes’ criteria^13^. Information about bone deformity, limb development, skin texture, condition of the digestive and urinary systems, function and appearance of the eyes and ears, and medication history was collected by inquiry and physical examination. MRI or temporal bone computed tomography (CT) was used to evaluate the middle and inner ear conditions whenever possible along with an auditory test. The condition of the eyes was evaluated by ophthalmologist with fundoscopy. Cardiac ultrasound were performed in cases with clinical suspicion of cardiac abnormalities. Results of genotypic and phenotypic analyses of family members were also collected if available.

### Statistics Analysis

Continuous variables were checked for normal distribution by SPSS version 21 software package (IBM, China). Normally distributed variables are described by the mean and SD, and nonnormally distributed variables are described by the median and interquartile range. Categorical variables are presented as a percentage. The OR and 95% CI were calculated by using an online OR calculator (http://www.hutchon.net/ConfidOR.htm/) (Bland and Altman, 2000). When OR > 5.0 and the 95% CI did not include 1.0, it was assumed that the prevalence of the variant in patients is significantly increased compared with the prevalence of controls.

## Results

### Patients Harboring *CHD7* RSVs

A total of 36 CHH (20 KS; 15 nCHH) out of 327 (11.01%) carried heterozygous *CHD7* gene RSVs (MAF <0.0001) (Supplementary Figure S1). All these patients complained of puberty absence and small testicular size [1.5 (1, 2.75)], and the percentage of cryptorchidism and concealed penis were 16.67% (6/36) and 5.56% (2/36), respectively. The mean diagnosis age was 19.75 ± 4.88 years. Among 13 patients who accepted the olfactory MRI, all patients had absence or hypoplasia of the olfactory bulb or (and) tract to different degrees. The results of hormone tests, the mean basal LH, FSH, and testosterone levels were consistent with hypogonadotropic hypogonadism, and the specific values are shown in Table 1.

**TABLE 1:** Basic information of 36 patients harboring *CHD7* rare variants.

Patients No.	Dignosis	Gender	Age of dignosis (year)	Olfactory MRI	Basal testicular size, left/right (cm)	Basal sex hormone examination			Typical HH			Variants			
						LH (basal) (mIU/L)	FSH (basal) (mIU/L)	T (basal) (nmoL/L)	Cryptorchidism or Concealed penis	Delayed puberty	Primary amenorrhoea	Nucleotide change	Protein change	Zygosity	Other variants
P1	KS	M	24	NE	NA	0.3	0.01	1.71	−	+	—	c.2347 C > T	p.Pro783Ser	Het	—
P2	nCHH	M	20	NE	3/3	0	0	1.1	−	+	—	c.6703 A > C	p.Lys2235Gln	Het	—
P3	KS	M	20	NE	2/2	0.3	1.1	0.8	−	+	—	c.7972-1G > C	—	Het	—
P4	KS	M	29	NE	5/5	0.1	0.34	3.05	−	+	—	c.6980 T > G	p.Val2327Gly	Het	—
P5	KS	M	26	1	0/3	0	0.2	1.03	Concealed penis	+	—	c.7083 G > C	p.Arg2361Ser	Het	—
P6	KS	M	14	2	1/1	0.06	1.8	1.02	−	+	—	c.3932 T > C	p.Ile1311Thr	Het	—
P7	KS	M	19	3	3/3	0.18	0.48	2.5	−	+	—	c.5227 C > T	p.Arg1743Cys	Het	
P8	nCHH	M	20	NE	6/2	2.6	0.3	1.96	Cryptorchidism (bilateral)	+	—	c.7170 T > G	p.Asp2390Glu	Het	—
P9	KS	M	24	3	2/2	0.04	0.7	−	Concealed penis Cryptorchidism (bilateral)	+	—	c.6353 A > G	p.Asn2118Ser	Het	—
P10	KS	M	22	NE	2/2	0.47	2.4	0.15	−	+	—	c.409 T > G	p.Ser137Ala	Het	—
P11	KS	F	18	NE	NA	1.34	2.5	39.6	−	+	−	c.2214 A > C	p.Glu738Asp	Het	—
P12	nCHH	M	20	NE	1/1	0.4	1.3	0.9	−	+	—	c.2831 G > A	p.Arg944His	Het	*WDR11*, p.Leu891Trp(Het)
P13	KS	M	20	NE	4/4	0.47	1.03	0.97	−	+	—	c.120 A > C	p.Gln40His	Het	—
P14	KS	M	19	NE	1/1	0	0.4	0.88	−	+	—	c.2658 C > T c.4516 G > A	p.Arg886Trp p.Gly1506Ser	Het	—
P15	nCHH	F	24	NE	NE	0	0	0.99	−	+	−	c.2831 G > A	p.Arg944His	Het	—
P16	KS	M	8	NE	1/1	0.04	1.2	0.49	Cryptorchidism (right)	+	—	c.1853 A > G	p.Asp618Gly	Het	*GNRH1*, p.Gly34Arg(Het)
P17	nCHH	M	19	NE	2/1	1.25	1.77	0.83	−	+	—	c.4516 G > A	p.Gly1506Ser	Het	—
P18	KS	M	14	1	2/2	0.06	1.5	1.36	−	+	—	c.2615 T > C	p.Ile872Thr	Het	—
P19	KS	M	28	NE	1/2	1.17	3.5	1.36	−	+	—	c.3752 G > T	p.Cys1251Phe	Het	—
P20	nCHH	M	22	NE	4/4	0.38	1.4	1.73	−	+	—	c.2835+1G > A/G	—	Het	—
P21	nCHH	M	15	NE	1/1	0	0.1	7.09	−	+	—	c.5210+3A > A/G	—	Het	—
P22	KS	M	12	3	1/1	0	0.3	0.07	Cryptorchidism (bilateral)	+	—	c.5095 A > G	p.Lys1699Glu	Het	*WDR11*, p.Val336Phe(Het)
P23	nCHH	M	21	NE	3/3	1.64	2	0.48	−	+	—	c.2662 A > G	p.Met888Val	Het	—
P24	nCHH	M	25	NE	0/0	0	0.07	2.85	Cryptorchidism (bilateral)	+	—	c.2219 A > G	p.Asp740Gly	Het	—
P25	KS	F	14	NE	—	0.1	1.1	0.4	−	+	—	c.6851 G > A	p.Arg2284Gln	Het	—
P26	KS	M	11	2	1/0.5	0.09	0.89	1.38	Cryptorchidism (bilateral)	+	—	c.7 G > A	p.Asp3Asn	Het	—
P27	nCHH	M	20	NE	2/2	0.42	0.8	0.46	−	+	—	c.2831 G > A	p.Arg944His	Het	—
P28	nCHH	M	30	NE	8/6	NA	NA	NA	−	+	—	c.2690 G > C	p.Arg897Pro	Het	—
P29	KS	M	19	2	1.5/1.5	0.22	1.3	0.89	−	+	—	c.8424 C > A	p.Asn2808Lys	Het	—
P30	KS	M	17	NE	1/1	0.01	0.44	0.62	−	+	—	c.2182 G > A	p.Asp728Asn	Het	—
P31	KS	F	18	3	NE	0	0.8	NA	−	+	+	c.6368 C > G	p.Ser2123Cys	Het	−
P32	KS	M	23	3	2/1	0	0.5	1.13	−	+	—	c.2214 A > C	p.Glu738Asp	Het	—
P33	KS	M	22	NE	2/3	1.66	2	0.12	−	+	—	c.7358 G > A	p.Ser2453Asn	Het	−
P34	KS	M	19	1	2/1	0	0.2	0.4	−	+	—	c.4516 G > A	p.Gly1506Ser	Het	−
P35	KS	M	19	1	1/1	0	0.4	0.97	−	+	—	c.120 A > C	p.Gln40His	Het	−
P36	KS	M	16	1	1/1	0	0.4	13.0	−	+	—	c.5227 C > T	p.Arg1743Cys	Het	−

nCHH, normosmic congenital hypogonadotropic hypogonadism; KS, Kallmann syndrome; 1, absence of olfactory bulb and tract, 2, hypoplasia of olfactory bulb and absence of olfactory tract, 3, hypoplasia of olfactory bulb and tract; M, male; F, female; NE, no evaluation; +, positive symptoms; −, negative symptoms; NA, not eavauation.

**FIGURE 2:**
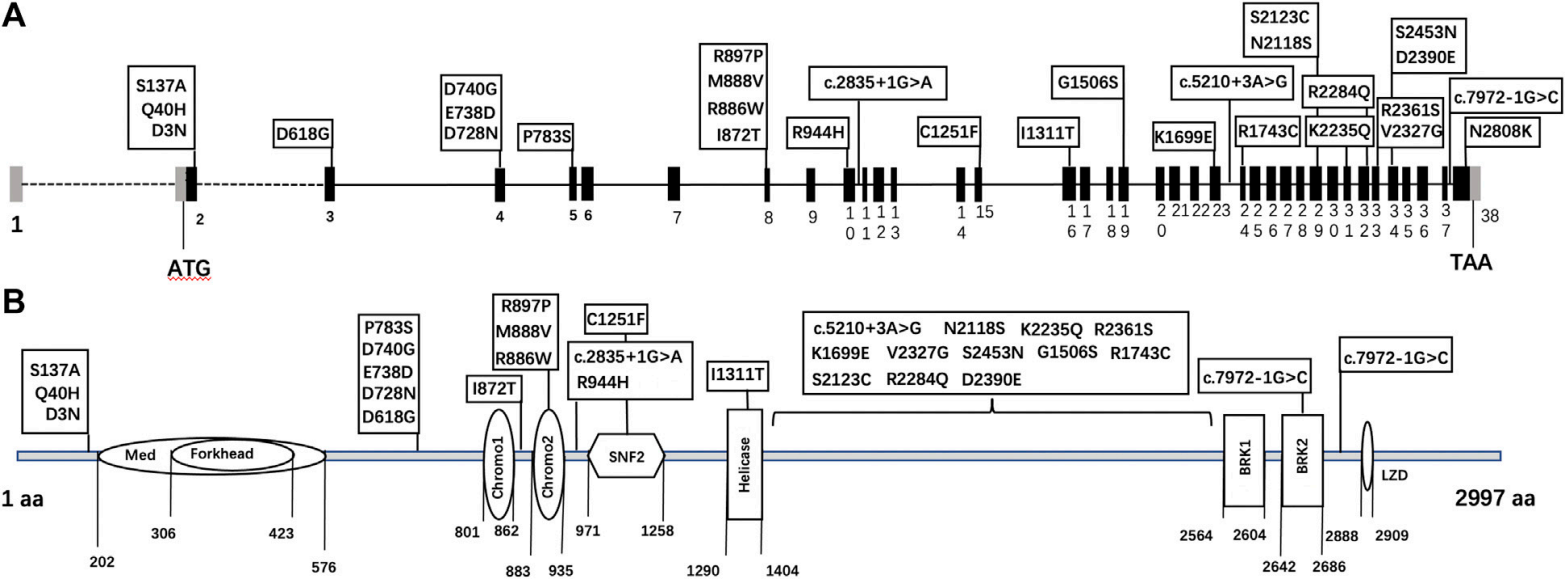
The schematic diagram of *CHD7* gene **(A)** and protein **(B)** structure. Black bars: coding exons; grey bars: non-coding sequences; various shapes in the protein diagram: functional domains of the CHD7 protein.

Thirty *CHD7* gene variants were detected, including three splice site (c.2835+1G > A, c.5210+3A > G, and c.7972-1G > C) and 27 missense variants (D3N, Q40H, S137A, D618G, D728N, E738D, D740G, P783S, I872T, R886W, M888V, R897P, R944H, C1251F, I1311T, G1506S, K1699E, R1743C, N2118S, S2123C, K2235Q, R2284Q, V2327G, R2361S, D2390E, S2453N, and N2808K) (Supplementary Figure S1). All variants were distributed throughout the *CHD7* gene and showed a certain cluster in exons 2, 4, and 8. Six variants were located in known functional domains of CHD7 protein: three (D740G, M888V, and R886W) in Chromo2, one (C1251F) in SNF2, one (I1311T) in Helicase, and one (c.7972-1G > C) in BRK2, and 12 variants clustered in a protein region that was not conserved and its function was unknown (Figure 2).

Particularly, each variant existed only in one proband, except for Q40H, R1743C and E738D presenting in two patients and G1506S in three patients. Three patients harbored other heterozygous missense gene variants besides *CHD7*: one patient with *GNRH1* (G34R in Patient 16) and two patients with *WDR11* (V336F in Patient 22 and L891W in Patient 12) (Table 1).

### Classification of Variants Based on the ACMG Guideline and Bioinformatics Analysis

We classified the 30 *CHD7* variants according to HGVS, then analyzed their pathogenicity based on ACMG criteria (Table 2). Besides R944H, which had OR < 5.0 and 95% CI included 1.0, the other variants were either absent in the gnomAD database or the corresponding OR ≥ 5.0 and 95% CI did not include 1.0. Among these 30 variants, 11 were recurrent and 19 were reported for the first time. Six variants existed in the ClinVar database: S137A, P783S and D2390E were reported in CHARGE syndrome patients; R944H both in CHARGE syndrome and HH patients; R2284Q had no specific disease description. The amino acid sites of five variants were reported in the *CHD7* variants database: three (c.5210+3 A > G, D728N, and R944H) had the same amino acid change and two (C1251F and R2284Q) mutated into a different one. Besides, four [D728N (Bartels et al., 2010), R886W (Marcos et al., 2014), D740G (Bilan et al., 2012), R944H (Bartels et al., 2010)] were reported by other researchers in CHH or CHARGE syndrome cohorts, and the detection rates were 1/642, 1/313, 1/50, and 1/642, respectively.

**TABLE 2:** Pathogenicity analysis of 29 *CHD7* variants.

Nucleotide change	Gnom AD (Allele frequency)		ClinVar (Interpreted condition)	CHD7 database (mutation ID)	Frequency in our Cohort		OR (95%CI)	Reference (PMID)		Recurrent or Novel	Parental origin	In Silico Analysis							ACMG criteria	Classification
												SIFT	Polyphen 2	SNPs & Co	Mutation assessor	MutPred	Splice site score calculation	Splice AI		
IVS10: c.2835+1G>A	—	—	—	—	0.0031	(1/327)	/	—	—	Novel	NA	—	—	—	—	—	+	+	PVS1; PM2; PP5; PP3	P
IVS23: c.5210+3A>G	—	—	—	M726	0.0031	(1/327)	/	—	—	—	NA	—	—	—	—	—	+	+	PVS1; PM2; PP5; PP3	P
IVS37: c.7972-1G>C	—	—	—	—	0.0031	(1/327)	/	—	—	Novel	NA	—	—	—	—	—	+	+	PVS; PM2; PP3	P
Ex2: c.409 T>G	0.000121	(34/280358)	CHARGE syndrome	—	0.0031	(1/327)	25.29 (3.45–285.30)	—	—	—	NA	−	−	+	+	+	—	—	PS4; PP5; PP3	LP
EX5: c.2347C>T	0.0000821	(23/280310)	CHARGE syndrome	—	0.0031	(1/327)	37.38 (5.03–277.63)	—	—	—	NA	−	−	+	+	+	—	—	PS4; PP5; PP3	LP
EX32: c.6851 G>A	0.000004	(1/249122)	not specified	Arg2284X (M49)	0.0031	(1/327)	764.17 (47.59–12243.89)	—	—	—	NA	−	+	+	+	+	—	—	PS4; PP5; PP3	LP
EX34: c.7170 T>G	0.0000291	(7/240698)	CHARGE syndrome		0.0031	(1/327)	105.47 (47.59–12243.89)	—	—	Novel	NA	−	+	+	+	+	—	—	PS4; PP5; PP3	LP
EX4: c2214 A>C	—	—	—	—	0.0062	(2/237)	/	—	—	—	NA	−	−	+	+	+	—	—	PM2; BP4	US1
EX10: c.2831 G>A	0.000623	(174/279338)	CHARGE syndrome; HH (likely behign)	M499	0.0031	(1/327)	4.92 (47.59–12243.89)	21158681	1/642 (CHARGE)	—	NA	−	−	+	+	+	—	—	PS4; BP6; PP3	US1
EX31: c.6703 A>C	5.66E-06	(1/176564)	—	—	0.0031	(2/327)	1086.55 (98.28–12013.78)	—	—	Novel	NA	−	−	+	+	+	—	—	PS4; BP4	US1
EX15: c.3752 G>T	—	—	—	Cys1251Arg (M1014)	0.0031	(1/327)	/	—	—	—	NA	+	+	+	+	+	—	—	PM2; PM5; PP3	US2
EX9: c.2656 C>T	—	—	—	—	0.0031	(1/327)	/	25077900	1/313 (HH)	—	NA	+	+	+	+	+	—	—	PM2; PP3; PP5	US2
EX19: c.4516 G>A	0.0000265	(7/264564)	—	—	0.0062	(2/327)	232.39 (48.09–1122.2)	—	—	Novel	NA	+	+	+	+	+	—	—	PS4; PP3	US2
EX4: c.2182 G>A	—	—	—	M473	0.0031	(1/327)	/	21158681	1/642 (CHARGE)	—	maternal	−	−	+	+	+	—	—	PS4; PP2; PP5; PP3	US2
EX31: c.6353 A>G	0.000111	(31/280350)	—	Asn2118Asp (M1199.Benign)	—	—	55.65 (48.09–1122.2)	—	—	—		−	−	+	+	+	—	—	—	US2
EX33: c.7083 G>C	7.35E-06	(2/272042)	—	—	0.0062	(2/327)	837.04 (117.55–5960.42)	—	—	Novel	NA	−	+	+	+	+	—	—	PS4; PP3	US2
EX34: c.7358 G>A	—	—	—	—	0.0031	(1/327)	/	—	—	Novel	Paternal	−	+	+	+	+	—	—	PM2; PP3	US2
EX38: c.8424 C>A	8.19E-06	(2/244278)	—	—	0.0031	(1/327)	374.66 (33.89–4142.13)	—	—	Novel	NA	−	−	+	+	+	—	—	PS4; PP3	US2
EX2: c.7 G>A	4.26E-06	(1/234534)	—	—	0.0031	(1/327)	719.42 (44.90–11526.92)	—	—	Novel	NA	−	+	+	+	+	—	—	PS4; PP3	US2
EX2: c.120 A>C	0.0000121	(3/248208)	—	—	0.0031	(1/327)	253.78 (26.33–2446.27)	—	—	Novel	NA	−	+	+	+	+	—	—	PS4; PP3	US2
EX9: c.2615 T>C	0.0000379	(9/237620)	—	—	0.0031	(1/327)	80.99 (10.34–641.07)	—	—	Novel	NA	−	+	+	+	+	—	—	PS4; PP3	US2
EX9: c.2662 A>G	0.0000252	(7/277580)	—	—	0.0031	(1/327)	121.64 (14.92–991.45)	—	—	Novel	NA	−	+	+	+	+	—	—	PS4; PP3	US2
EX3: c.1853 A>G	—	—	—	—	0.0031	(1/327)	/	—	—	Novel	paternal	−	+	+	+	+	—	—	PM2; PP3	US2
EX4: c.2219 A>G	—	—	—	—	0.0031	(1/327)	/	22033296	1/50 (CHARGE)	Novel	NA	−	+	+	+	+	—	—	PM2; PM5; PP3	US2
EX9: c.2690 G>C	—	—	—	—	0.0031	(1/327)	/	—	—	Novel	NA	−	−	+	+	+	—	—	PM2; PP3	US2
EX16: c.3932 T>C	—	—	—	—	0.0031	(1/327)	/	—	—	Novel	maternal	+	+	+	+	+	—	—	PM2; PP3	US2
EX23: c.5095 A>G	—	—	—	—	0.0031	(1/327)	/	—	—	Novel	maternal	+	−	−	+	+	—	—	PM2; PP3	US2
EX31: c.6368 C>G	—	—	—	—	0.0031	(1/327)	/	—	—	Novel	maternal	+	−	+	+	+	—	—	PM2; PP5; PP3	US2
EX33: c.6980 T>G	—	—	—	—	0.0031	(1/327)	/	—	—	Novel	NA	+	+	+	+	+	—	—	PM2; PP3	US2
EX24: c5227 C>T	—	—	—	—	0.0031	(1/327)	/	—	—	Novel	NA	+	+	+	+	+	—	—	PM2; PP3	US2

*, pathogenic ≥3; P, pathogenic; LP, likely pathogenic; US1, uncertain significance with paradoxical evidence; US2, uncertain significance without enough evidence; US, uncertain significance; NA, not available.

**FIGURE 3:**
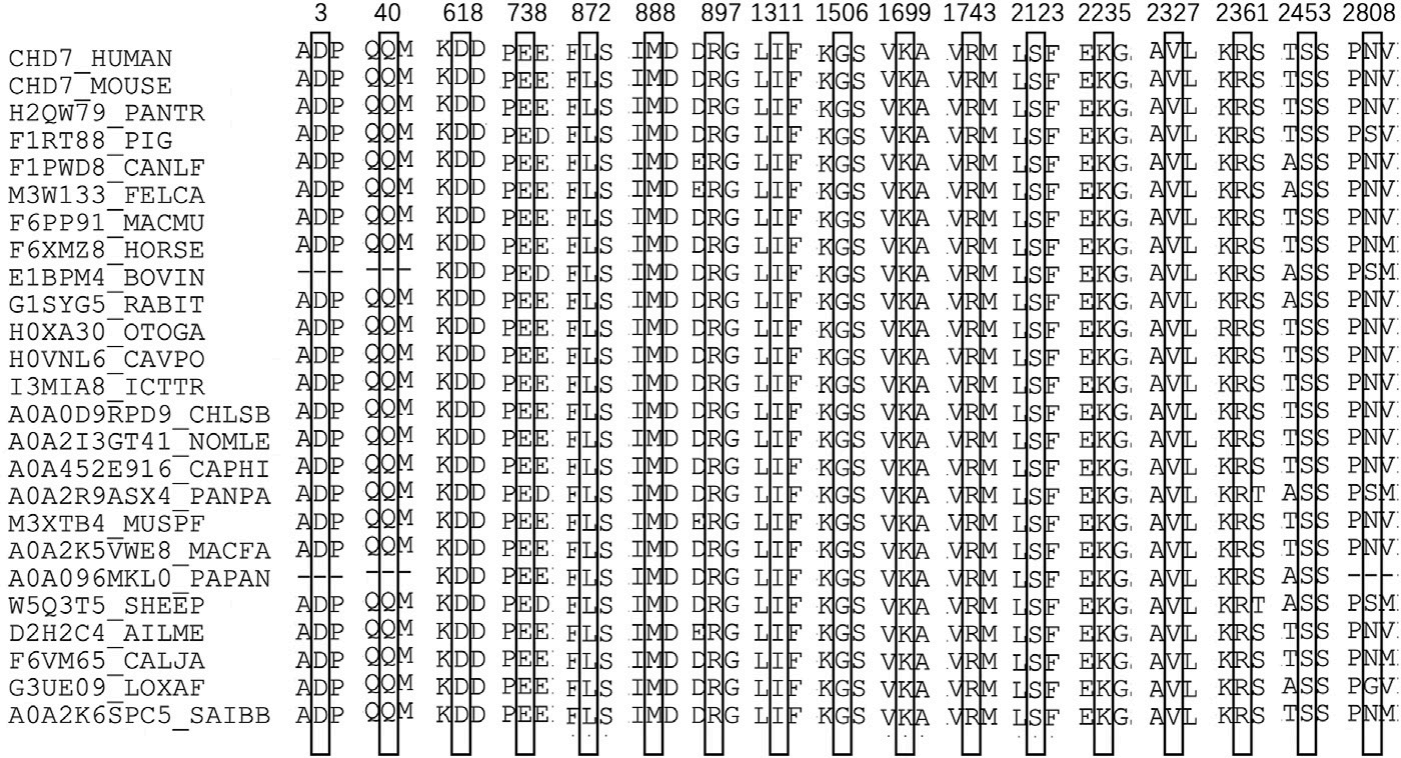
Sequence alignment of CHD7 proteins from 25 different species. The numbers and boxes indicate the corresponding changed amino acid identified in this study. The cysteine residue in black numbers and boxes at each position of CHD7 protein is conserved across 25 species.

**FIGURE 4:**
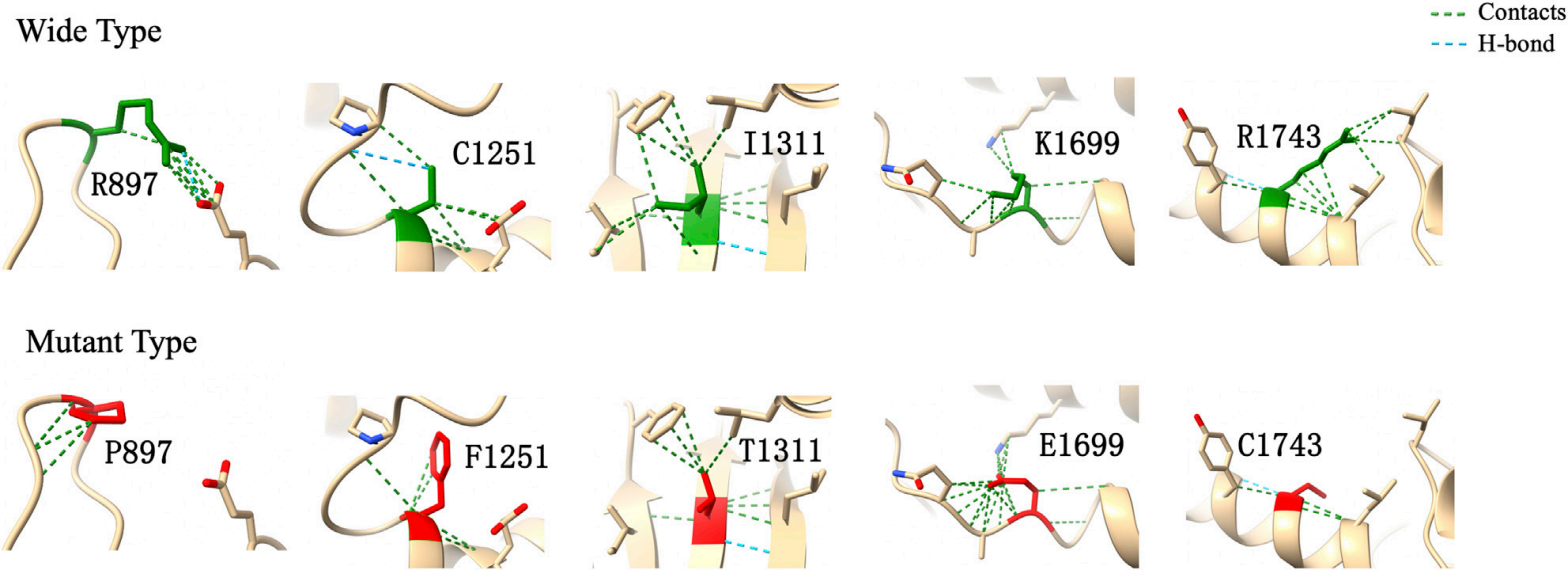
Three-dimensional structural modeling of wild and mutant CHD7 protein. These structural models were predicted by using SwissModel and visualized in ChimeraX. The models above and below represent the wild type (green) and mutant forms (red), respectively; the specific changes are labelled.

In computational pathogenicity prediction, firstly, the results of in silico analyses showed that, except for E738D and K2235Q (less than three tools defined these as pathogenic) with BP4 evidence, the remaining missense variants and three splicing site variants all were pathogenic and showed PP3 evidence. Secondly, focusing on 17 novel missense variants, alignment results indicated that except for N2808K, the other 16 wild type residues at a specific site were highly conserved across 25 different species (Figure 3). Thirdly, as three-dimensional models of R897P, I1311T, C1251F, R1743C, and K1699E (built by SwissModel) are available, we can see the direct harmful effect of a single amino acid change in the tertiary structure of the protein. The size, charge, and hydrophobicity of all five residues changed at the specific site: for R897P, the acidic amino acid changed into a nonpolar aliphatic one. Seven old contacts and 1 hydrogen bond (H-bond) broke, which accompanied with 6 new contacts and 1 clashes formed; for C1251F, the neutral polar amino acid changed to an aromatic one, besides, 1 old contacts, 1 clashes (unfavorable interactions where atoms are too close together) and 1 H-bond lost; for I1311T, a neutral polar one replaced the nonpolar aliphatic amino acid, and 3 old contacts broke; for K1699E, 8 new contracts formed; for R1743C, 7 contacts lost (Figure 4). Based on these findings, 3/30 (10.00%) variants were classified as P variants, 4/30 (13.33%) were LP, 3/30 (10.00%) were US1, and 20/30 (66.67%) were US2.

None of the second variant could be classified as the digenic pathogenic one according to the existing evidence. *GNRH1* variants were inherited in an autosomal recessive manner; therefore, a direct pathogenic effect was impossible. *WDR11* variants, which showed an autosomal dominant pattern of inheritance, had V336F classified as US1 and L891W as US2. Owing to the lack of experimental evidence, it is necessary to study whether these variants contribute to disease pathogenesis in a synergistic manner.

### Clinical Evaluation and Genotype-Phenotype Correlation

Twenty-three patients (16 KS and seven nCHH; 21 male and two female) harboring RSVs accepted detailed clinical evaluation, including two with P variant, two with LP variant, two harboring US1, and 17 with US2 (Table 3). Most of them (65.22%; 15/23) had extra-gonad phenotypes; CHARGE or CHARGE-like symptoms accounted for 80.0% (13/15) cases and other *CHD7-*related symptoms were seen in 53.3% (8/15) cases. All CHARGE or CHARGE-like features, except for rhombencephalic anomalies and malformation of mediastinal viscera, occurred in our patients; hearing loss and high myopia were the most frequent ones (5/10; 50%). Patient 21(P21 in Table 3) was re-diagnosed as having typical CHARGE syndrome based on three major and six minor diagnostic items. Other *CHD7-*related symptoms included high arched palate (50%; 4/8), spinal malformation (25%; 2/8), digestive system dysfunction (25%; 2/8), secondary hypothyroidism (12.5%; 1/8), six fingers in the left hand (12.5%; 1/8), inguinal hernia (12.5%; 1/8), micrognathism (12.5%; 1/8), widened palpebral fissure (12.5%; 1/8), epicanthus (12.5%; 1/8), and short philtrum fish mouth (12.5%; 1/8).

**TABLE 3:** *CHD7*-related characteristics analysis in 23 CHH patients harboring *CHD7* rare variants.

Patients	Dignosis	Sex	Nucleotide Change	Other variants	CHARGE syndrome related symptoms		Others
					Major	Minor or CHARGE-like symptoms	
Patient with P variants							
P20	nCHH	M	c.2835+1G > G	—	UA	Deafness;	Scoliosis
						Abnormal external ear	Digestive system
						Intellectual Disability	disfuction
						Malformation of mediastinal organs (heart)	—
							—
P21	nCHH	M	c.5210+3A > G	—	Chanal atresia; Coloboma; semicircular canal anomalies	High Myopia Neuro sensory deafness	High arched palate
						Facial asymmetry	
						Abnormal external ear	Scoliosis
						Intellectual Disability	—
						Malformation of mediastinal organs (heart)	—
						Microphthalmia	—
Patient with LP variants							
P25	KS	F	c.6851 G > A	—	—	Deafness	High palate
						Abnormal external ear	
						Growth hormone deficiency	
P8	nCHH	M	c.7170 T > G	—	—	Intellectual Disability	—
Patients with US1 variants							
P32	KS	M	c.2214 A > C	—	—	—	—
P2	nCHH	M	c.6703 A > C	—	—	—	—
Patients with US2 variants							
P14	KS	M	c.2656 C > T	—	—	—	Inguinal hernia
			c.4516 G > A				
P34	KS	M	c.4516 G > A	—	—	—	High palate; polysyndactyly (left hand)
P33	KS	M	c.7358 G > A	—	—	Growth hormone deficiency	—
P35	KS	M	c.120 A > C	—	—	Micrognathism	High palate
						Widened palpebral fissure	
						Epicanthus	
						Short philtrum fish mouth	
P16	KS	M	c.1853 A > G	GNRH1, p.Gly34Arg	—	—	—
P24	nCHH	M	c.2219 A > G	—	—	High	—
						Myopia	
P6	KS	M	c.3932 T > C	—	—	—	Secondary
							hypothyroidism
P31	KS	F	c.6368 C > G	—	—	—	—
P7	KS	M	c.5227 C > T	—	—	—	—
P36	KS	M	c.5227 C > T	—	—	—	—
P4	KS	M	c.6980 T > G	—	—	Deafness	—
						High Myopia	
P18	KS	M	c.2615 T > C	—	—	Malformation of mediastinal organs (heart)	—
						Growth hormone deficiency	
						High Myopia	
P17	nCHH	M	c.4516 G > A	—	—	Deafness	—
						Abnormal external ear	
						Growth hormone deficiency	
P5	KS	M	c.7083 G > C	—	—	—	—
P23	nCHH	M	c.2662 A > G	—	—	High	Digestive system
						Myopia	disfuction
P29	KS	M	c.8424 C > A	—	—	—	—
P26	KS	M	c. 7 G > A	—	—	Deafness	—

KS, kallmann syndrome; nCHH, normosmic congenital hypogonadotropic hypogonadism; M, male; F, female; UA, unavailable.

Occurrence of extragonadal symptoms correlated with the pathogenicity of variants. All CHH patients with P or LP variant manifested CHARGE-related symptoms or (and) other CHD7-related manifestations besides CHH. For patients carrying US1 and US2 variants, the frequency of CHARGE-related symptoms or others was 0% (2/2) and 64.71% (11/17), respectively.

## Discussion

We identified 30 types of *CHD7* RSVs with a detection rate of 11.01% [8.11% in nCHH (12/148) and 13.41% in KS (24/179)] in our series of 327 CHH patients. P or LP variants accounted for 23.33% (7/30) cases. Previous studies on the pathogenic role of *CHD7* RSVs mainly focused on CHARGE syndrome, In 2008, Kim et al. (2008) first showed that Chd7 mRNA expressed in the CHH/KS-relevant organs of rats (including migratory and post-migratory GnRH neuron, olfactory bulb, pituitary, and hypothalamus), and the pathogenic variants can exist in both nCHH and KS without CHARGE-related phenotype. Thus, the triple correlation among *CHD7*, CHH, and CHARGE started to gain importance. Our *CHD7* detection rate was consistent with that reported in previous four studies, which explored *CHD7* variants in CHH patients, with detection rates of 16% (18/116) (Xu et al., 2018), 16% (8/50) (Gonçalves et al., 2019), 10.2% (18/177) (Li et al., 2020), and 6% (6/101)^9^.

Our variants distributed throughout the *CHD7* gene and protein region, and tended to cluster around exons 2, 4, and 8 of the gene model and three regions without known functional domain of the protein model, whereas pathogenic or likely pathogenic RSVs showed no “hot-spot” tendency. To further explore the potential function of the unknown protein regions (amino acids 1–202; 576–801; 1,404–2,564), we looked them up in the InterPro website and found no conserved regions. This may be an evidence for the hypothesis that *CHD7* variants in humans can lead to a continuous phenotype spectrum, and CHH is just a milder manifestation than CHARGE syndrome (Kim et al., 2008). The report byBergman et al. (2012) also supports this hypothesis. In their CHARGE syndrome patients, pathogenic missense mutations were mainly found in the middle of the *CHD7* gene, where functional domains clustered.

We annotated the variants manually according to the ACMG guideline and further classified US variants into US1 and US2, which may be suggestive for variant classification in the future. Most of the variants (66.67%; 20/30) were classified as US. Four variants (E738D, A2225T, K2235Q, and R944H) belonged to US1 (need to be further confirmed by functional experiments) and 20 variants (G1506S, N2118S, R2361S, N2808K, D3N, Q40H, I872T, S2453N, D618G, D740G, R897P, I1311T, K1699E, S2123C, V2327G, R1743C, D728N, C1251F, R886W, and M888) belonged to US2 (reclassification may be required with additional global evidence obtained through studies in the future).

One patient (P21 in Table 3) was re-diagnosed as having CHARGE syndrome in our CHH series. Similarly, the study by Xu et al. reclassified 3 out of 17 patients. Except for rhombencephalic anomalies and malformation of the mediastinal viscera, all CHARGE syndrome diagnostic items were noted in our CHH patients with *CHD7* variants, and hearing loss (5/10; 50%) and high myopia (5/10; 50%) were the most common ones. However, in the study by Wu et al., hearing loss (6/18, 33.3%) and ear deformities (3/18, 16.7%), the two diagnostic CHARGE features, were significantly frequent in patients with *CHD7* variants. As for other symptoms besides CHH and CHARGE, we first reported polysyndactyly, inguinal hernia, small chin and short philtrum fish mouth in CHH patients with *CHD7* variants, and all these phenotypes existed in the same patient (P35 in Table 3), who carried the variant Q40H.

No consensus on the genotype-phenotype correlation exists. In our study, we observed a correlation between variant pathogenicity and extragonadal symptoms. Extragonadal symptoms were present more commonly in patients with P or LP variants (4/4, 100.0%) than in patients with US1 (0/2, 0%) or US2 (11/17, 64.71%) variants. In 2017, Xu et al. (2018) first evaluated CHARGE syndrome in 166 CHH patients, and detailed phenotyping in 17 patients revealed that 80% (4/5) of patients with P or LP variants showed multiple CHARGE features versus 8% (1/12) with nonpathogenic (US, B, and LB) variants. However, in studies by Jongmans et al. (2006) and Bergman et al. (2011), no genotype-phenotype correlation existed in CHARGE syndrome patients. Due to the small sample of patients with P and LP variants and the lack of awareness of the true classification of US variants, we could not ascertain the difference between each subgroup. Therefore, even though specific clinical manifestations can provide us with information on genetic hits, this finding needs to be interpreted with caution.

The main strengths of our study are as follows: ① This study systematically analyzed genotype, phenotype, and their correlation in a series of CHH patients with *CHD7* gene variants; ② 19 novel variants were reported to expand the genotype spectrum; ③ Variants were classified according to the ACMG guideline, and the US type variants were further classified into US1 and US2, which provide detailed evidence for future studies; ④ Our study showed a genotype-phenotype correlation tendency, namely P or LP RSVs were more likely to cause syndromic CHH.

Besides, there are also some limitations in our study that need to be mentioned. ① Owing to partial information provided by the probands’ family, evidence for the genotype-phenotype co-segregation was unavailable, which could elaborate the contribution of *CHD7* variants to these phenotypes besides CHH; ② This comprehensive clinical analysis was retrospective, and loss of follow-up existed; therefore, the prevalence of phenotypes besides CHH may be underestimated in CHH patients with *CHD7* variants. In future studies, it would be necessary to collect information on the probands’ family blood tests and perform comprehensive physical examination.

## Conclusion

19 novel *CHD7* variants reported herein expand the existing variant spectrum. P or LP RSVs are more likely to cause syndromic CHH. For CHH patients carrying *CHD7* RSVs, early detailed genotyping and phenotyping can help clinical diagnosis and therapy.

## Data Availability

The datasets presented in this study can be found in online repositories. The names of the repository/repositories and accession number(s) can be found in the article/Supplementary Material.
