# Supplementary figures and images for: Classification of CHD7 Rare Variants in Chinese Congenital Hypogonadotropic Hypogonadism Patients and Analysis of Their Clinical Characteristics
Source: Front Genet. 2022 Jan 3;12:770680. doi: 10.3389/fgene.2021.770680 (PMC8762265; doi:10.3389/fgene.2021.770680)

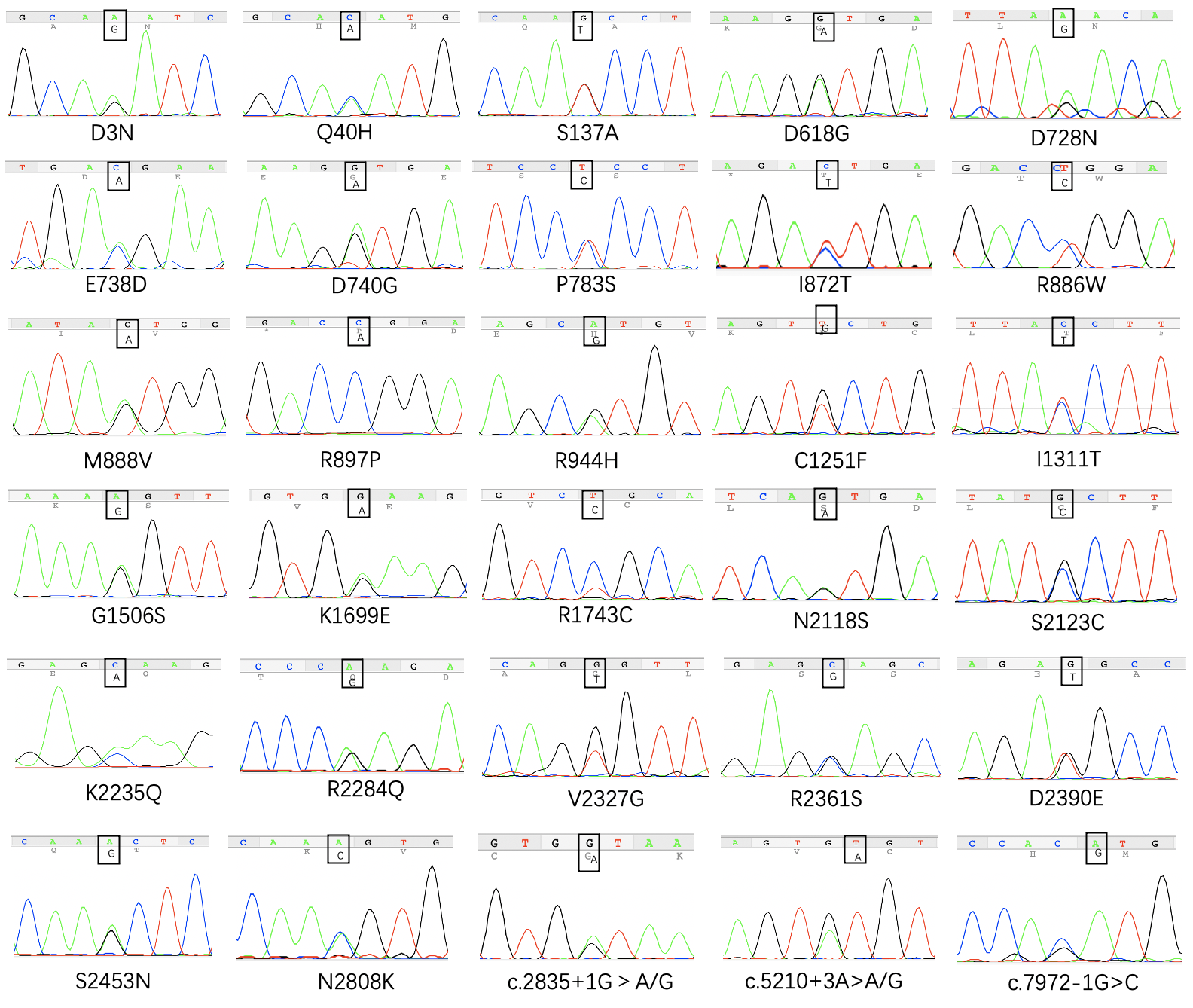

Supplement: Supplementary file 1 [file Image1.TIFF]
